# The Visionary Journey of Dr. Brij Nandan Singh Walia

**DOI:** 10.7759/cureus.88867

**Published:** 2025-07-27

**Authors:** Harvinder Kaur, Divjot Kaur, Anil Bhalla

**Affiliations:** 1 Child Growth and Anthropology Unit, Department of Pediatrics, Postgraduate Institute of Medical Education and Research, Chandigarh, IND

**Keywords:** "historical vignette", legacy, pediatrics, pgimer, public health

## Abstract

Dr. Brij Nandan Singh Walia is a towering figure in Indian medicine, whose visionary leadership and pioneering contributions have left an enduring imprint on pediatric care and medical education. A luminary in every sense, Dr. Walia continues to inspire generations of healthcare professionals with his dedication, compassion, and transformative vision. Born in 1933 in Hoshiarpur, Punjab, Dr. Walia’s passion for science and medicine was evident from an early age. He pursued his MBBS from Mahatma Gandhi Memorial Medical College, Indore, followed by a Doctorate of Medicine (MD) and a Diploma in Child Health. This strong academic foundation laid the groundwork for a lifetime of medical innovation and institutional leadership. Dr. Walia’s most profound impact was at the Postgraduate Institute of Medical Education and Research (PGIMER), Chandigarh, where he served in multiple capacities as Associate Professor, Head of the Department of Pediatrics, and ultimately as Director of the institute. As the founding architect of the Advanced Pediatric Centre at PGIMER, Dr. Walia transformed the landscape of child healthcare in India, establishing a model of excellence that integrates clinical care, research, and education. He was also instrumental in securing the recognition of pediatrics as a distinct specialty within the MBBS curriculum. A staunch proponent of global best practices, he facilitated international training for faculty and introduced institutional reforms that positioned PGIMER among the foremost medical institutions in the world. As Director, his dynamic stewardship brought about sweeping advancements in infrastructure, research, and patient care. Beyond the hospital walls, his commitment to community health, equity in education, and social welfare has uplifted countless lives. Through the Bhai Jaita Ji Foundation, he supported rural students and championed initiatives for underprivileged communities. He also advocated for institutions dedicated to the care of the disabled. Dr. Walia’s legacy is not merely institutional-it is transformational, exemplifying how one individual’s vision, compassion, and unwavering resolve can redefine the future of healthcare.

## Introduction and background

Dr. Brij Nandan Singh Walia is a distinguished pediatrician and a visionary leader whose contributions to the field of medicine and child welfare have left an indelible mark on healthcare in India. As the founding architect of the Advanced Pediatric Centre at the Postgraduate Institute of Medical Education and Research (PGIMER), Dr. Walia not only established a robust framework for pediatric care but also played a pivotal role in enhancing the department's reputation on an international scale. He took the initiative to train his staff at world-renowned institutions, ensuring that they received the highest quality of education and training experience.

Under his leadership, pediatrics got recognized as a separate specialty in MBBS, elevating its importance within the medical community. Dr. Walia’s dedication to excellence led to his appointment as Director of PGIMER, and he fostered a culture of work prioritization, devoting significant time and effort to advancing PGIMER, including the construction of major hospital facilities that continue to benefit countless patients. Renowned for his visionary leadership and steadfast commitment to medical excellence, he played a pivotal role in elevating PGIMER to international standards. Under his guidance, the institution saw a significant increase in research endeavors, with faculty members obtaining prestigious grants and publishing influential works in renowned journals.

Dr. Walia’s bio sketch narrates a journey of unwavering dedication, innovative breakthroughs, and a mission to enhance the lives of the youngest in the society.

## Review

Early life and education

**Figure 1:**
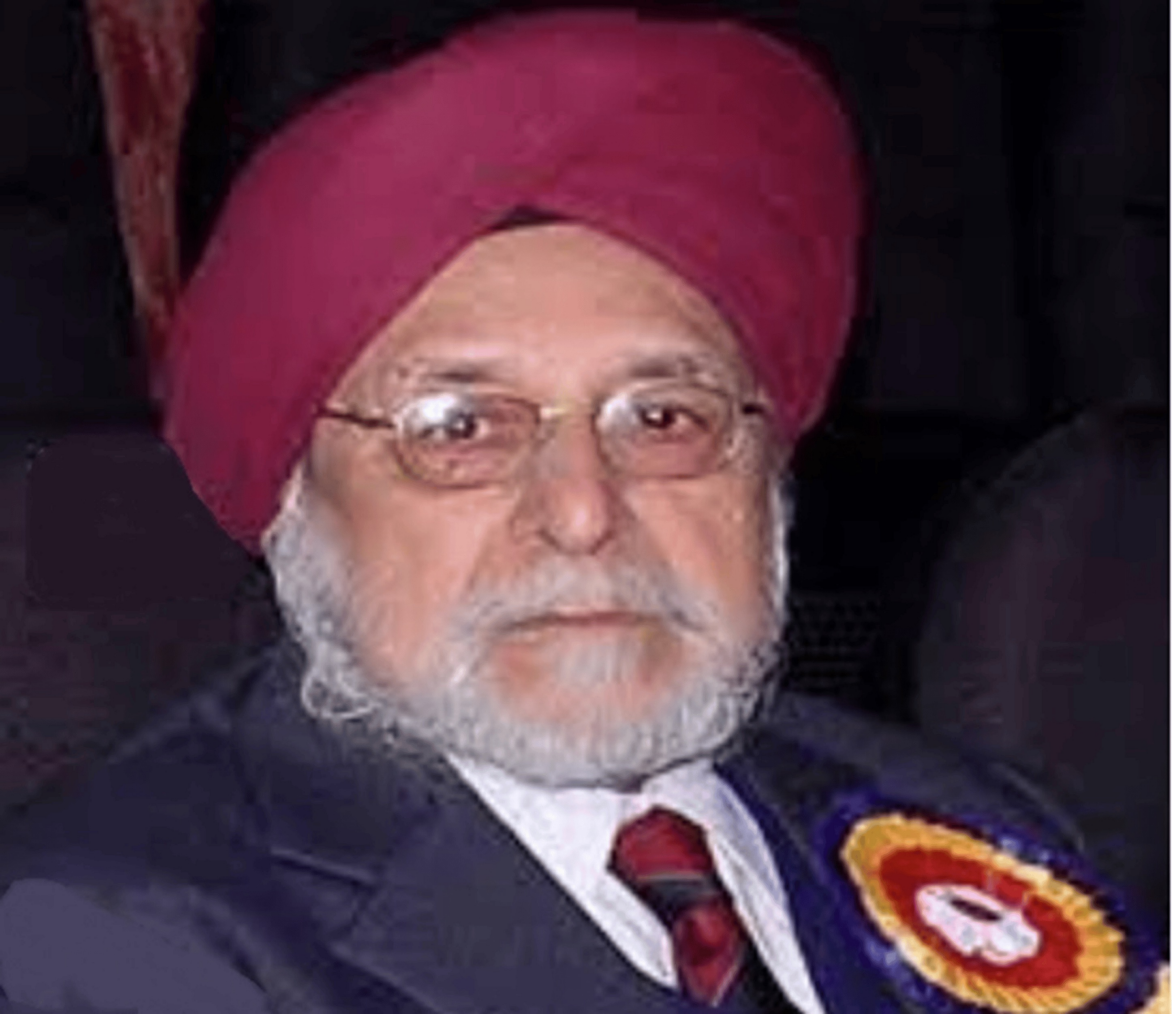
Dr. Brij Nandan Singh Walia Image used with permission.

Dr. Brij Nandan Singh Walia, born on June 23, 1933, at Hoshiarpur, Punjab, to Mr. Rajender Singh and Ms. Gursharan Kaur, grew up in a family that placed a high value on education (Figure 1). His father, an office superintendent, ensured that all his children attended reputable schools and became as good at English as possible. As the eldest of four siblings, Dr. Walia developed a strong interest in science and medicine from a young age, inspired by his maternal uncle, Dr. Majheil Singh Pal, a well-known physician of his time.

He completed his primary education at Sohanlal High School in Lahore and then, from Harkot Butler School in Delhi, and finished his secondary education at DAV School, Delhi, where he focused on biology. He then attended Hindu College in Delhi for his 12th grade.

Dr. Walia earned his MBBS degree from Mahatma Gandhi Memorial Medical College, Indore, in 1954 and began his house job in medicine in the same year. He did his Doctorate of Medicine (MD) (1955-1957), followed by a Diploma in Child Health (DCH) in 1958.

Professional journey

Dr. Walia was initially offered a position as a lecturer in Medicine by a prominent professor in that field. However, he declined the offer, feeling a deep commitment to pediatric care that could not allow him to leave the children [1]. Instead, he continued his work in pediatrics and joined All India Institute of Medical Sciences (AIIMS), Delhi, as Registrar in 1958 and was appointed as an Assistant Professor in Pediatrics in 1961. Later, in 1965, he joined PGIMER, Chandigarh, as Associate Professor and Head of the Department of Pediatrics. Initially allotted 50 beds, he successfully negotiated for an increased capacity of 80 beds, comprising 50 for pediatric medicine and 30 for pediatric surgery. Dr. Walia floated the idea of a separate pediatric center for medicine and surgery.

His clinical expertise and compassionate approach earned him respect from peers and patients alike. However, it was his organizational skills and vision for systemic improvement that facilitated his rise into administrative roles. Dr. Walia was offered a fellowship by the British Pediatric Association, funded by Heinz and Nuffield Corporation, for one year in 1968. He spent five months studying undergraduate education and the broader field of public health in Britain at the University of Newcastle under Dr. F.J.W. Miller, followed by six months at the Institute of Child Health, London.

Leadership at PGIMER

Dr. Walia's appointment as the Director of PGIMER on February 8, 1991, marked a pivotal moment for the institution. His leadership ushered in a comprehensive transformation that reshaped PGIMER's future. Dr. Walia believed that effective leadership was not just about authority; it was about inspiring others, fostering collaboration, and driving collective success.

During his 52-month tenure, PGIMER achieved several significant milestones in patient care, staff training, and overall welfare. He oversaw the expansion of departments, the addition of new buildings, and the integration of advanced cutting-edge technologies relating to patient care and treatment. A highlight of his vision was the realization of the “Advanced Pediatrics Centre,” a testament to his commitment to enhancing pediatric care.

One of Dr. Walia’s primary goals was to broaden PGIMER’s educational offerings and research capabilities. Each department was thoughtfully designed not only to improve patient care but also to position PGIMER as a hub for innovative medical research. He prioritized staff training by facilitating fellowships at prominent foreign institutions, ensuring that his team was equipped with the latest knowledge and skills.

Dr. Walia’s approach to leadership extended beyond structural changes. He implemented mentorship programs for young doctors and faculty, fostering a culture of continuous learning and professional development. By nurturing the next generation of healthcare leaders, he ensured that PGIMER would remain at the forefront of medical education.

His key values-transparency, employee welfare, reforms, and discipline-were integral to his leadership style, creating an environment where collaboration and innovation thrived. Through his visionary guidance, Dr. Walia left an indelible mark on PGIMER, setting the stage for its future successes.

Realization of the dream, setting up of the Advanced Pediatric Center (APC)

Dr. Walia’s vision for the APC was ignited during his fellowship at the Institute of Child Health at Great Ormond Street, London, in 1968. During this time, he had the opportunity to visit five leading pediatric centers in Bristol, Liverpool, Glasgow, Edinburgh, and London. Working alongside Dr. David Morley and Dr. June Lloyd at Great Ormond Street, Dr. Walia conceived the idea of establishing a center of excellence for Community Pediatrics and a center for tertiary pediatric care in Chandigarh.

Over the years, the development of the APC became an all-consuming passion for him. He drew inspiration from the design of the Pediatric Center in Bern, Switzerland, which featured four distinct towers: one for faculty offices, another for research labs, a third for inpatients, and a fourth for outpatients. This layout facilitated uninterrupted work hours for the faculty. The neonatal unit's design was adapted from a model in Manila, while the center's exterior, including a garden and a statue symbolizing mother and child, was inspired by designs from Stockholm.

Securing funding for his envisioned project proved to be a prolonged and difficult journey. Despite numerous efforts and rejections, he remained persistent. Eventually, during the Sixth Five-Year Plan, a revised proposal was approved, paving the way for the establishment of an independent center with many super specialties of pediatrics, a 300-bed capacity, and a sanctioned budget of ₹20 crore.

Construction of the APC finally commenced in 1992. The emergency block was completed first, and the full structure of the APC was completed in 1997, two years after Dr. Walia’s superannuation, and was inaugurated by Dr. S.D. Sharma, then President of India. Thus, Dr. Walia’s long-cherished dream of creating a world-class pediatric center was ultimately realized, marking a significant milestone in pediatric care in India.

Innovations in medical education and staff training

A significant part of Dr. Walia's legacy at PGIMER is his commitment to medical education reform. It was not until 1992 that pediatrics was a separate subject at the MBBS examination level. In the year 1992, the Medical Council of India thought of reviewing the curriculum for MBBS, which had been designed in 1962. A committee was formed in which Dr. Walia was also invited to represent the case of pediatrics. His advocacy for pediatrics was so compelling and was also supported by the IMA president and UNICEF chief, that Pediatrics was included as a separate subject in the final MBBS level. He was also invited to join the committee for planning services for maternal and child health during the fifth and sixth five-year plans. Under the fifth plan, initiatives such as measles vaccination, oral rehydration solutions (ORS), and postgraduate nursing education in neonatal and cardiac nursing were introduced. He prepared a postgraduate nursing program, and a National Institute of Nursing Education (NINE) was approved as a new institute at PGIMER.

One of Dr. Walia's most notable achievements was his dedication to the professional development of PGIMER's faculty. He actively encouraged and supported faculty members in pursuing fellowships and training programs at prestigious institutions abroad. By providing them with opportunities to learn from international experts, Dr. Walia empowered his faculty to acquire cutting-edge skills and knowledge, ultimately benefiting patients and the medical community as a whole. He met with the Health Secretary of the Government of India to reform the fellowship allocation system, resulting in 150 fellowships - 12 annually for PGIMER - to facilitate skill development across specialties.

Team development was always a priority for Dr. Walia, and he ensured that training and skill development were emphasized across all specialties at PGIMER. Nursing and paramedical staff were also sent for training in intensive care units, and perfusionists received specialized training for cardiac surgery. In addition to these initiatives, annual training programs for educators in teaching technology were also organized, and staff members received management training from visiting faculty at the Indian Institute of Technology (IIT) Delhi. His efforts ensured that PGIMER graduates were not only well-versed in medical theory but also equipped with the practical skills necessary for real-world challenges.

To address accommodation needs for patients’ attendants, he initiated the construction of a Sarai (inn) with 400 beds. He also planned and implemented hostels for nurses, senior residents, and working women, as well as a sports complex and modern housing for PGIMER staff. A crèche for children was relocated to a more equipped facility, and a benevolent fund was established to assist those in need. Dr. Walia’s commitment to social responsibility highlighted his belief that medical institutions should serve the community, making PGIMER a model for community engagement and health promotion through partnerships with local organizations and government bodies.

After retiring, he joined the Bhai Jaita Ji Foundation to support students from socio-economically compromised backgrounds in rural Punjab, helping them prepare for competitive entrance exams to enter top engineering and medical institutes. More than 350 students have been admitted to professional colleges under this program. His influence also extended to public health, as he launched outreach programs for underprivileged communities, focusing on preventive care and health education. He spearheaded the movement for an Institution for handicapped children in Chandigarh, India, which was ultimately created in 1998 and started much-needed training of staff in the management of handicapped children. 

Legacy and recognition

**Figure 2:**
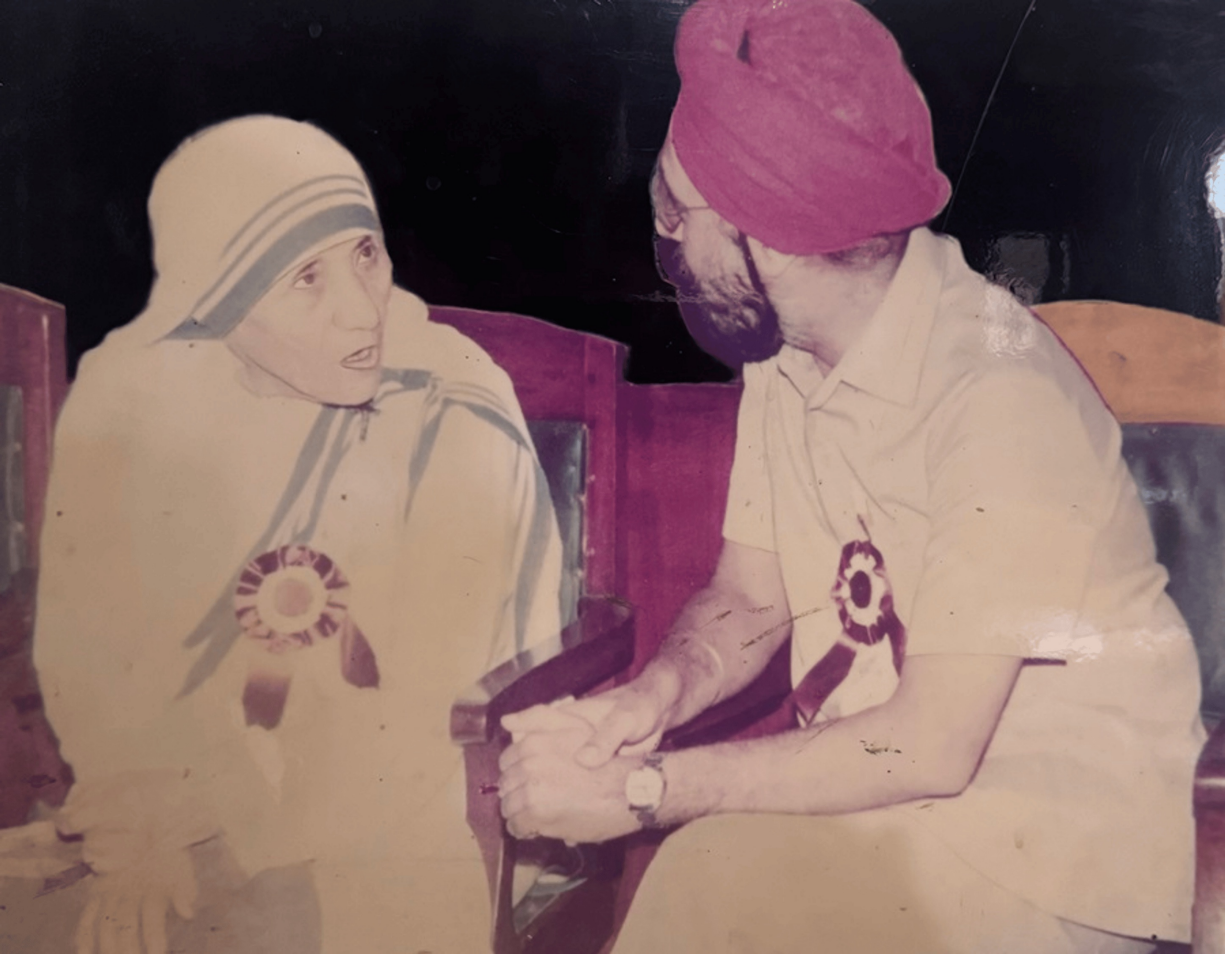
Dr. Brij Nandan Singh Walia with Mother Teresa at the IAP Annual Conference (1983) Image used with permission.

Dr. Walia's tenure at PGIMER has been marked by numerous accolades and recognition from national and international bodies. His contributions to medicine and education have earned him a place among the leading figures in Indian healthcare. He has been invited to speak at numerous conferences and has published extensively in reputable medical journals [2], sharing his insights on leadership, education, and research (Figure 2). He was awarded founder membership of the Royal College of Pediatrics and Child Health, London, in 1997.

As he continues to lead PGIMER into the future, Dr. Walia remains a beacon of excellence in the medical field. His unwavering commitment to leadership, innovation, and community service sets a high standard for medical professionals everywhere. His vision for PGIMER not only reflects his dedication to improving healthcare but also his belief in the transformative power of education and research.

## Conclusions

Dr. Brij Nandan Singh Walia’s journey exemplifies the profound impact of visionary leadership in healthcare. His innovative approaches, dedication to education, and commitment to community health have redefined medical leadership. As a pivotal figure at PGIMER, he established a legacy that will inspire future generations of healthcare professionals to pursue excellence and make meaningful societal contributions. His relentless pursuit of advancing pediatric care and medical education has left an enduring mark on both the institute and the broader medical community. Dr. Walia's story is one of triumph over challenges, highlighting the significant influence one individual can have on the future of healthcare facilities and services.
